# Immunoprofiling of Colitis-associated and Sporadic Colorectal Cancer and its Clinical Significance

**DOI:** 10.1038/s41598-019-42986-1

**Published:** 2019-05-02

**Authors:** Jae Seung Soh, Su In Jo, Hyejin Lee, Eun-ju Do, Sung Wook Hwang, Sang Hyoung Park, Byong Duk Ye, Jeong-Sik Byeon, Suk-Kyun Yang, Ji Hun Kim, Dong-Hoon Yang, Sang-Yeob Kim, Seung-Jae Myung

**Affiliations:** 1 Department of Internal Medicine, Hallym University Sacred Heart Hospital, University of Hallym College of Medicine, Anyang, Korea; 2 Asan Institute for Life Sciences, Asan Medical Center, Seoul, Korea; 3 Department of Gastroenterology, Asan Medical Center, University of Ulsan College of Medicine, Seoul, Korea; 4 Department of Pathology, Asan Medical Center, University of Ulsan College of Medicine, Seoul, Korea; 5 Department of Convergence Medicine, University of Ulsan College of Medicine, Asan Medical Center, Seoul, Korea

**Keywords:** Cancer microenvironment, Colon cancer

## Abstract

Immunoprofiling is useful for predicting prognosis in various malignancies and provides targets for immunotherapy. Quantitative multispectral imaging system, which allows simultaneous detection of multiple immune markers, is a novel method for examining the tumor immune environment. We compared the expression levels of various surface markers in immune cells between colitis-associated cancer (CAC) and sporadic colorectal cancer (CRC) and evaluated the clinical usefulness of immunoprofiling in CRC. Tumor specimens from 24 CAC patients and 48 sporadic CRC patients, matched by age, sex, and tumor location to CAC, were included in the analysis. The expression levels of CD3, CD8, Foxp3, and programmed death-ligand 1 (PD-L1) in immune cells at the invasive margins of tumor tissues were evaluated by quantitative multispectral imaging. The CAC group had significantly less levels of cells expressing CD3, CD8, Foxp3, or PD-L1 (all, *p* < 0.01). In the CAC group, patients whose immune cells had high expression of CD3^+^ and CD8^+^ had better overall survival. The immune profiling patterns of CAC patients were significantly distinct from those of sporadic CRC patients, suggesting that CAC and sporadic CRC have distinct disease phenotypes. Immunoprofiling can be helpful for evaluation of clinical prognosis in CAC.

## Introduction

Currently, the most common system for classifying the extent of tumor progression is the TNM classification established by the American Joint Committee on Cancer^1^. However, evaluating malignant disease only with the TNM system is often insufficient, because patients with the same histological tumor stage tend to show different clinical outcomes. The critical roles of immune function and tumor microenvironment have been recognized, and immunotherapy is becoming an integral component of cancer therapy^2^. Immunoprofiling is useful for predicting prognosis, including disease-free survival (DFS) and overall survival (OS), and for providing targets for immunotherapy^3^. In lung cancer and melanoma, high expression levels of CD3, CD4, and CD8 in immune cells were correlated with good prognosis^4,5^; also, patients with high number of programmed death-ligand 1 (PD-L1)^+^ cells have shown good response to treatment using anti-PD-1 antibody^6,7^. In colorectal cancer (CRC) patients, high expression levels of immune markers including CD3, CD8, and CD45RO were associated with better DFS and lower tumor recurrence^8–10^.

Patients with long-standing inflammatory bowel disease (IBD) including ulcerative colitis (UC) and colonic Crohn’s disease (CD) have higher risk of colitis-associated cancer (CAC)^11^. Chronic relapsing intestinal damage caused by severe inflammation promotes cancer development and alters various kinds of immune cells and cytokines^12^. The innate and adaptive immune systems related to chronic inflammation play both pathogenic and protective roles in tumor microenvironment. Therefore, the inflammation-dysplasia-carcinoma pathway of IBD-associated cancer may have different immune reactions from those of the adenoma-carcinoma pathway of sporadic CRC. Until now, however, there have been no reports comparing the surface marker expression levels in immune cells in CAC and sporadic CRC.

Recently, a novel system of quantitative multispectral immunohistochemical (IHC) imaging system has been developed for studying immune cells^13^. Whereas conventional IHC staining method could not simultaneously detect multiple markers and lacked automated quantification, the quantitative multispectral imaging system enables identification of different biomarkers in the same tissue section by using tyramide signal amplification, which simultaneous staining of up to six IHC target markers^14^. Also, automated image analysis softwares have been developed to accurately quantify immune markers by offering improved sensitivity for marker detection, reduced antibody consumption, and excellent resolution with a lower background^15^.

We aimed to compare the expression levels of various immune-related surface markers in immune cells between CAC patients and sporadic CRC patients, and to evaluate the clinical usefulness of immunoprofiling in CRC.

## Results

### Baseline characteristics of the study population

A total of 24 patients with CAC and 48 patients with sporadic CRC were enrolled in this study. The median age of the CAC group at diagnosis was 43 years (range, 27–67 years) and that of the sporadic CRC group was 45 years (range, 29–77 years) (Table 1). Men were 54.2%, and tumors were mostly located in the rectum, followed by the left colon (splenic flexure to sigmoid colon) and right colon (cecum to transverse colon) in both groups. At the time of diagnosis, stage IV in CAC was 33.3% (eight patients) and the rest were stages I–III (16 patients). In contrast, stage IV in sporadic CRC was 8.4% (four patients) and the rest were stages I–III (44 patients) (*p* = 0.015). The surgical procedures for the two groups were different: total colectomy was carried out in 54.2% of the CAC group, whereas lower anterior resection was carried out in 79.1% of the sporadic group (*p* < 0.001). The median follow-up durations for CAC and sporadic groups were 41 months and 43 months, respectively. Recurrence rates were not significantly different between the two groups. Cancer-related death was significantly higher in CAC than in sporadic CRC (41.7% *vs*. 14.6%, *p* = 0.018). The pathologic characteristics including the median diameter of operation specimen, lymphovascular invasion, and microsatellite instability (MSI) between both groups were not significantly different.

**Table 1:** Baseline characteristics between colitis-associated cancer patients and sporadic colorectal cancer patients.

Variables	CAC (n = 24)	Sporadic (n = 48)	*p*-value
Age, years, median (range)	43 (27–67)	45 (29–77)	0.357
Male sex, n (%)	13 (54.2)	26 (54.2)	1.000
Location of tumors			0.916
Right-sided, n (%)	4 (16.7)	7 (14.6)	
Left-sided, n (%)	5 (20.8)	12 (25.0)	
Rectum, n (%)	15 (62.5)	29 (60.4)	
CEA level at the diagnosis of cancer			0.529
≥5, n (%)	6 (25.0)	8 (16.7)	
<5, n (%)	18 (75.0)	40 (83.3)	
Stage (AJCC 7^th^)			**0.015**
I, n (%)	5 (20.8)	5 (10.4)	
II, n (%)	6 (25.0)	16 (33.3)	
III, n (%)	5 (20.8)	23 (47.9)	
IV, n (%)	8 (33.3)	4 (8.4)	
Operation methods			**<0.001**
Lower anterior resection, n (%)	5 (20.8)	38 (79.1)	
Right hemicolectomy, n (%)	1 (4.2)	7 (14.6)	
Total colectomy, n (%)	13 (54.2)	3 (6.3)	
Tumor excision or biopsy with palliative ileostomy, n (%)	5 (20.8)	0 (0.0)	
Follow-up months, median (range)	41 (3–176)	43 (1–125)	0.302
Recurrence during follow-up periods, n (%)	5 (20.8)	8 (16.7)	0.749
Cancer-related death, n (%)	10 (41.7)	7 (14.6)	**0.018**
Pathologic features	CAC (n = 19)	Sporadic (n = 48)	
Median diameter of operation specimen, cm (range)	5.0 (1.0–16.0)	4.9 (0.7–9.0)	0.352
Lymphovascular invasion, n (%)	8 (42.1)	20 (35.4)	0.610
MSI-high, n (%)	3 (15.8)	8 (16.7)	0.741

CAC, colitis-associated cancer; MSI, microsatellite instability.

### Characteristics of colitis-associated cancer

Of the 24 patients, eight were CD and 16 were UC. Sixteen patients (66.7%) had disease duration of more than 10 years and 11 patients (45.8%) showed moderate-to-severe disease activity at the time of CAC diagnosis. Immunosuppressive agents including azathioprine and 6-mercaptopurine were used in eight patients (33.3%), and anti-TNF agents were used in three patients (12.5%). Immunosuppressive agents were administered for ≥1 year and anti-TNF agents, which were all infliximab, were infused for ≥6 months before CAC diagnosis. Among the eight CD patients, six patients (75.0%) were diagnosed with rectal cancer associated with chronic perianal fistula.

### Comparison of the quantification of immune cells between colitis-associated cancer and sporadic colorectal cancer

**Figure 1:**
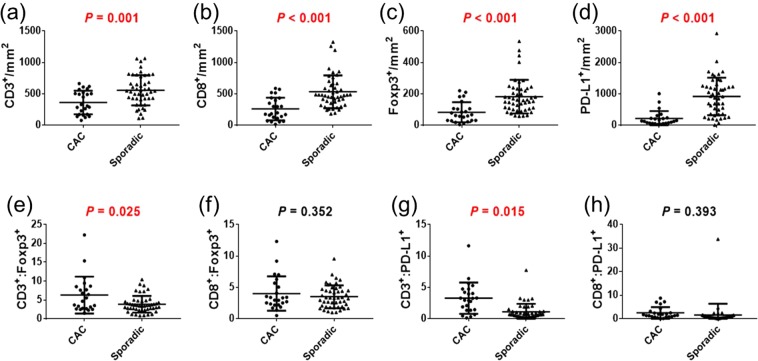
(**a**–**d**) Comparison of immune cell quantification between colitis-associated cancer (CAC) and sporadic colorectal cancer (CRC) according to the phenotyping method. (**e**–**h**) Ratios of immune cells in CAC and sporadic CRC patients.

**Figure 2:**
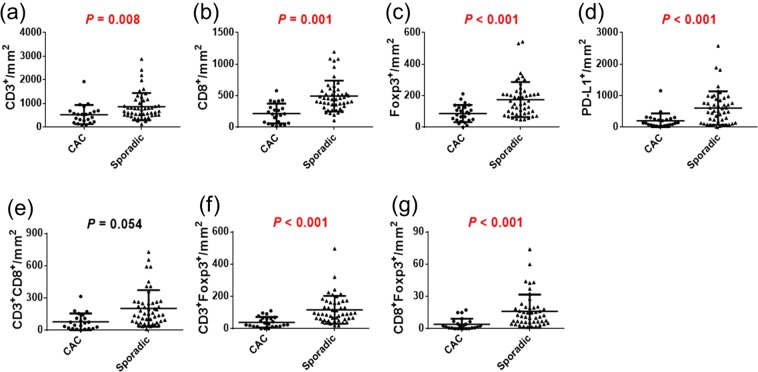
(**a**–**d**) Comparison of immune cell quantification between CAC and sporadic CRC according to the double positivity scoring method. (**e**–**g**) Comparison of co-expressing cells between CAC and sporadic CRC patients.

**Figure 3:**
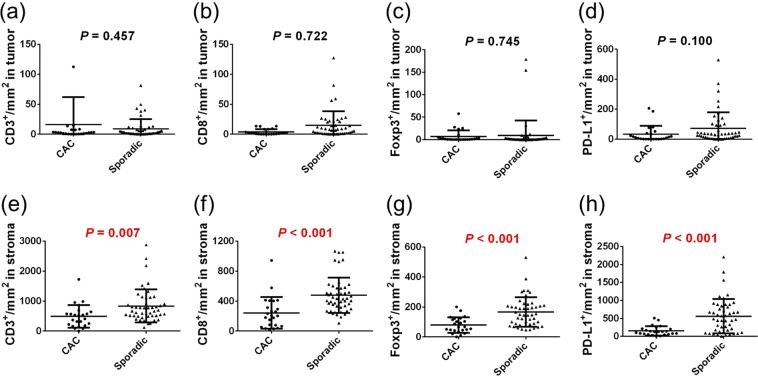
(**a**–**d**) Comparison of cells expressing immune markers in the tumor between CAC and sporadic CRC patients. (**e**–**h**) Comparison of cells expressing immune markers in the stroma region between CAC and sporadic CRC patients.

We evaluated the quantification of immune cells by phenotyping and double positivity scoring analysis. The counts per mm^2^ of CD3^+^, CD8^+^, Foxp3^+^, and PD-L1^+^ cells in CAC patients were significantly lower than those in sporadic cancer patients according to both the phenotyping method (Fig. 1) and the double positivity scoring method (Fig. 2) (all, *p* < 0.01). As for the ratio of immune cells, the CD3^+^-to-Foxp3^+^ ratio and the CD3^+^-to-PD-L1^+^ ratio of CAC patients were significantly higher than those of sporadic cancer patients. In the analysis carried out with the double positivity scoring method, the densities of CD3^+^Foxp3^+^ and CD8^+^Foxp3^+^ cells were significantly lower in CAC patients than in sporadic cancer patients (all, *p* < 0.001). The immune cells were divided into cells in the tumors and cells in the stroma regions according to positive or negative staining of CK. The cell showing double positivity for CK and CD3 was defined as CD3^+^ T cell infiltrated in the tumor, whereas the cell expressing CK^−^ and CD3^+^ was defined as CD3^+^ T cell in the stroma region. As for the double positivity scoring of CK and other immune markers, the densities of cells expressing immune markers in the tumor were not significantly different between CAC and sporadic cancer (Fig. 3). However, the densities of cells expressing immune markers in the stroma region were significantly lower in CAC patients than those in sporadic cancer patients (all, *p* < 0.01). In a subgroup analysis of the immune cells between CAC patients and stage-matched sporadic CRC patients in stages I – III, densities of all immune markers of CAC patients were significantly lower than those of sporadic cancer patients (Supplementary Table 1).

### Comparison of the quantification of immune cells according to factors in colitis-associated cancer

Table 2 shows the expression levels of immune markers according to various clinicopathological factors in CAC patients. The counts per mm^2^ of CD3^+^, CD8^+^, Foxp3^+^, and PD-L1^+^ cells did not show significant differences related to factors such as disease type (CD or UC), age, tumor location, CEA level, disease activity, and lymphovascular invasion. The densities of CD3^+^, CD8^+^, and Foxp3^+^ cells of patients in TNM stages I-III at the time of CAC diagnosis were higher than those in stage IV. The densities of CD8^+^ and Foxp3^+^ cells in patients using anti-TNF agents were lower than those of non-users. Use of steroids and immunosuppressive agents at the time of CAC diagnosis did not show significant difference in terms of immune cells densities. In CAC patients, the densities of all four kinds of immune cells were not significantly different according to the MSI status. Sporadic CRC patients showed similar results in that there were no significant differences in the densities of immune cells according to the MSI status.

**Table 2:** Comparison of immune cells according to various factors in patients with colitis-associated cancer.

Disease	CD (n = 8)	UC (n = 16)	*p*-value
CD3, mm^2^, mean (SD)	371 (205)	358 (185)	0.818
CD8, mm^2^, mean (SD)	166 (101)	306 (194)	0.153
Foxp3, mm^2^, mean (SD)	64 (67)	92 (63)	0.214
PD-L1, mm^2^, mean (SD)	304 (319)	168 (182)	0.192
Age	<43.3 (n = 14)	≥43.3 (n = 10)	*p*-value
CD3, mm^2^, mean (SD)	415 (172)	289 (192)	0.105
CD8, mm^2^, mean (SD)	282 (195)	228 (160)	0.476
Foxp3, mm^2^, mean (SD)	77 (69)	90 (60)	0.633
PD-L1, mm^2^, mean (SD)	274 (273)	130 (156)	0.148
Location of tumor	Left colon (n = 20)	Right colon (n = 4)	*p*-value
CD3, mm^2^, mean (SD)	337 (189)	490 (126)	0.157
CD8, mm^2^, mean (SD)	249 (176)	312 (215)	0.525
Foxp3, mm^2^, mean (SD)	80 (61)	96 (87)	0.794
PD-L1, mm^2^, mean (SD)	223 (260)	169 (72)	0.525
CEA	<5 (n = 18)	≥5 (n = 6)	*p*-value
CD3, mm^2^, mean (SD)	357 (179)	379 (229)	0.820
CD8, mm^2^, mean (SD)	273 (183)	219 (178)	0.537
Foxp3, mm^2^, mean (SD)	82 (62)	85 (77)	0.923
PD-L1, mm^2^, mean (SD)	220 (258)	195 (186)	0.974
Stage	Stage I–III (n = 16)	Stage IV (n = 8)	*p*-value
CD3, mm^2^, mean (SD)	436 (170)	214 (124)	**0.007**
CD8, mm^2^, mean (SD)	339 (160)	100 (89)	**<0.001**
Foxp3, mm^2^, mean (SD)	105 (64)	37 (32)	**0.007**
PD-L1, mm^2^, mean (SD)	189 (172)	263 (345)	0.487
Disease activity	Mild (n = 13)	Moderate to severe (n = 11)	*p*-value
CD3, mm^2^, mean (SD)	342 (196)	385 (183)	0.733
CD8, mm^2^, mean (SD)	277 (184)	239 (181)	0.733
Foxp3, mm^2^, mean (SD)	95 (54)	69 (75)	0.093
PD-L1, mm^2^, mean (SD)	136 (102)	306 (318)	0.167
Steroids	Use (n = 3)	No use (n = 21)	*p*-value
CD3, mm^2^, mean (SD)	434 (229)	352 (185)	0.401
CD8, mm^2^, mean (SD)	347 (206)	247 (178)	0.354
Foxp3, mm^2^, mean (SD)	166 (86)	71 (53)	0.052
PD-L1, mm^2^, mean (SD)	118 (89)	227 (251)	0.561
Immunosuppressive agents	Use (n = 8)	No use (n = 16)	*p*-value
CD3, mm^2^, mean (SD)	345 (200)	371 (187)	0.881
CD8, mm^2^, mean (SD)	184 (133)	297 (191)	0.264
Foxp3, mm^2^, mean (SD)	71 (69)	89 (63)	0.528
PD-L1, mm^2^, mean (SD)	296 (325)	172 (180)	0.320
Anti-TNF agents	Use (n = 3)	No use (n = 21)	*p*-value
CD3, mm^2^, mean (SD)	191 (64)	387 (187)	0.101
CD8, mm^2^, mean (SD)	64 (48)	287 (174)	**0.023**
Foxp3, mm^2^, mean (SD)	22 (15)	91 (64)	**0.041**
PD-L1, mm^2^, mean (SD)	557 (440)	165 (161)	0.082
Lymphovascular invasion	Positive (n = 8)	Negative (n = 11)	*p*-value
CD3, mm^2^, mean (SD)	385 (182)	415 (199)	0.657
CD8, mm^2^, mean (SD)	328 (185)	288 (174)	0.717
Foxp3, mm^2^, mean (SD)	99 (64)	90 (70)	0.717
PD-L1, mm^2^, mean (SD)	193 (232)	253 (266)	0.442
MSI status	MSI-high (n = 3)	MSS (n = 16)	*p*-value
CD3, mm^2^, mean (SD)	435 (180)	396 (194)	0.875
CD8, mm^2^, mean (SD)	379 (196)	291 (173)	0.359
Foxp3, mm^2^, mean (SD)	88 (13)	95 (72)	0.712
PD-L1, mm^2^, mean (SD)	153 (18)	242 (269)	1.000

CD, Crohn’s disease; UC, ulcerative colitis; MSI, microsatellite instability; MSS, microsatellite stable.

### Comparison of overall survival according to the immune cells in colitis-associated cancer and sporadic colorectal cancer

**Figure 4:**
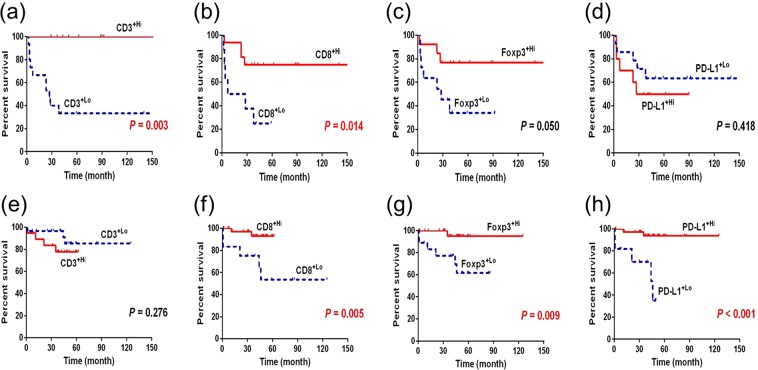
(**a**–**d**) Overall survival of CAC patients using Kaplan-Meier method according to the expression of immune cells. (**e**–**h**) Overall survival of sporadic CRC patients using Kaplan-Meier method according to the expression of immune markers.

Figure 4 shows the difference in overall survival using the Kaplan-Meier method between the high and low expression of immune cells. The high- and low-expression of immune markers was defined according to the cut-off value calculated in the ROC curve according to mortality. In the CAC group, patients whose immune cells had high expression of CD3 and CD8 had better overall survival. Those with high expression of Foxp3 tended to have better OS, albeit without statistical significance. Expression levels of PD-L1 did not show significant difference in terms of OS. In the sporadic CRC group, high expression of CD8, Foxp3, and PD-L1 was associated with better OS, whereas CD3 did not show significant difference.

### Comparison of immune markers between conventional and multiplexed IHC staining

Conventional and multiplexed IHC staining showed no statistically significant difference for any immune markers expressed as H-score between the two groups (Table 3). We obtained a similar result when we analyzed CAC and sporadic cancer separately.

**Table 3:** Comparison of immune cells between conventional and multiplexed immunohistochemical staining.

Variables	Conventional IHC	Multiplexed IHC	95% CI	*p*-value
CK, mean (SD)	176.5 (23.5)	175.2 (26.8)	−9.6, 13.7	0.858
CD3, mean (SD)	28.2 (14.3)	29.1 (14.6)	−6.6, 3.0	0.829
CD8, mean (SD)	20.5 (10.3)	21.1 (11.1)	−3.6, 2.6	0.845
PD-L1, mean (SD)	11.9 (12.4)	13.3 (13.9)	−8.7, 1.6	0.715
Foxp3, mean (SD)	5.9 (9.5)	5.4 (6.9)	−1.9, 4.0	0.841

IHC, immunohistochemistry; CI, confidence interval; SD, standard deviation.

## Discussion

Our present study describes, for the first time, the immunoprofiling of CAC compared with sporadic CRC using a quantitative multispectral imaging system. Most CRC arises from polyps through adenomatous change, whereas CAC arises from flat dysplastic mucosa. Accordingly, we hypothesized that CAC would show different levels of immune marker expression compared with that of sporadic CRC. T cells are associated with inflammation, cancer development, and tumor progression through adaptive immune system. Because CAC is associated with chronic inflammation, the densities of immune cells, especially T cells, are expected to be higher than those of sporadic CRC. The only study so far that evaluated T cells in colitis-associated dysplasia compared to sporadic CRC using a quantified IHC method was conducted by Michael-Robinson *et al*.^16^. In that report, the counts of CD3^+^ and CD8^+^ cells were significantly higher in colitis-associated lesions than in sporadic cancer, which is contrary to our study. The authors included patients with dysplasia as well as cancer and evaluated malignant tissues only in microsatellite-stable status, whereas our study enrolled only the patients diagnosed with CRC. Also, our CAC group included more patients with late-stage than did the sporadic CRC group and different operation methods were performed in the two groups. We found that the number of CD3^+^, CD8^+^, Foxp3^+^, and PD-L1^+^ cells were lower in the CAC group. Due to the discrepancy in baseline characteristics of the two groups, the specific roles of immune cells in the initiation and progression of CAC are yet to be fully delineated.

A possible explanation for the low densities of immune cells in CAC is the use of therapeutic agents such as steroids, immunosuppressive agents, and anti-TNF agents in IBD patients. Carcinogenic mechanisms associated with these therapeutic agents impair immune surveillance and reduce the number and function of immune cells^17^. A study from China showed that compared with placebo, anti-TNF therapy in patients with rheumatoid arthritis significantly altered the levels of regulatory T cells and suppressed effector T cells^18^. Our results showed that patients using anti-TNF at the time of CAC diagnosis had lower densities of CD8^+^ and Foxp3^+^ cells compared with non-users. In addition, 13 patients (54.2%) and nine patients (37.5%) had histories of steroid and immunosuppressive agents use, respectively. Further studies are needed to explain the relationship between the use of therapeutic agents and the immune system, but our data suggest that low density of immune cells in CAC may be associated with the use of therapeutic agents. Also, patients treated with anti-TNF agents may need to undergo a more intensive degree of cancer surveillance program to detect dysplasia and cancer.

Compared with sporadic CRC patients, CAC patients are younger, have multiple cancerous lesion, and are diagnosed with advanced stage at presentation, and severe histologic inflammation in UC patients is an independent risk factor for CAC^19^. Accordingly, our CAC patients showed less favorable clinical outcomes and more frequently underwent total colectomy. We also included more advanced stage patients in the CAC group, which led to more deaths in this group during follow-up. Total colectomy was performed on 12 out of 16 UC patients (75.0%), and six among eight CD patients (75.0%) had perianal fistula associated with CAC, which was suggested as a distinct feature of Korean CD patients compared with Western patients^20^. These features may be associated with the levels of immune cells in CAC patients, which was significantly different from those in sporadic CRC patients.

Previous studies on the role of immune cells in CRC demonstrated that T cell markers were associated with clinical outcomes. Patients with high densities of CD8^+^ cells had earlier stages of tumors and less tumor recurrence than did patients with low densities of CD8^+^ cells^9^. In addition, high densities of CD3^+^ and CD8^+^cells in the tumor center and invasive margins were correlated with better DFS and OS rates^10^. High density of Foxp3^+^ cells in tumor tissues was associated with improved survival in CRC patients^21^. These findings are in line with our results that in the CAC group, patients with high levels of CD3^+^, CD8^+^, and Foxp3^+^ cells had lower cancer stages than did patients with low levels of these cells. Also, high levels of CD3^+^ and CD8^+^ cells in CAC and that of CD8^+^, Foxp3^+^, and PD-L1^+^ in sporadic CRC were correlated with better OS. Thus, our data show that immune markers may be useful as a prognostic factor in patients with CAC as well as CRC.

PD-L1 is a transmembrane protein expressed on various solid tumors and immune cells, and it plays an important role in blocking T-cell activity by binding PD-1. Previous cancer studies including CRC patients showed that PD-L1 expression correlated with poor prognosis as well as aggressive activities^22^. However, several other reports also indicated a lack of significant association between PD-L1 expression and prognosis^23^ or even improved survival according to PD-L1 expression^24^. Our study is the first to apply multispectral IHC including PD-L1 to analyze the immune environment of IBD-associated cancer. In our study, high levels of PD-L1^+^ cells was associated with better OS in sporadic CRC patients, but not in CAC patients, indicating that PD-L1, as well as T cells, could be used as a prognostic factor in a disease-specific manner. High level of PD-L1^+^ cells may allow the use of anti-PD-L1 antibodies as therapeutic agents in select cases of CRC.

We used a novel quantitative multispectral imaging method by employing the Opal Multiplex IHC kit and Vectra imaging system to examine the microenvironment of CAC and sporadic CRC. The most notable advantage of this system is being able to perform IHC staining of multiple markers on single 4-micron sections of FFPE tumor samples. We were thus able to clearly detect four immune cells and cytokeratin simultaneously. In addition, this method enabled us quantitatively analyze the expression of immune markers. Several studies using this multispectral imaging system reported that the system enables highly accurate analysis of the immune environment in various tumors^25,26^. Feng *et al*.^26^ found no significant difference between using the percentage of immune cells or the count of immune cells per mm^2^ in image analysis of melanoma samples; therefore, we analyzed the immune markers with the count of immune cells per mm^2^ in this study. Although previous studies carried out cell analysis using only phenotyping, we additionally examined the expression of immune cells by using the double positivity scoring method. There was no significant difference between the two methods when analyzing the levels of immune cells. In addition, the double positivity scoring method had an advantage of being able to analyze immune cells in a quantitative manner. For the first time in literature, we report a comparative analysis of conventional and multiplexed IHC staining, and show that multiplexed IHC staining showed similar results to conventional IHC staining for all tested markers. This result further validates the newly developed multispectral imaging system for evaluating immune cells.

Our study has the following limitations. First, the samples were all from a single center and collected in a retrospective manner, which may add selection bias to our results. The small number (n = 24) of enrolled CAC patients may have resulted in selection bias. Second, correlation between the levels of immune cells and the use of immunosuppressive agents or anti-TNF agents was unclear. The densities of immune cells were not significantly different according to use of immunosuppressive agents in CAC patients. Although the densities of CD8^+^ and Foxp3^+^ cells in patients using anti-TNF agents was significantly lower than those of non-users, there is a possibility of sampling bias because only three patients used anti-TNF agents. Third, the sporadic CRC group was not matched with the CAC group in terms of cancer stage, with the latter including more patients in stage IV. In order to conduct survival analysis associated with immune markers, cancer stage matching would be needed.

In conclusion, the immunoprofiling pattern of CAC was different from that of sporadic CRC, suggesting that CAC and sporadic CRC have distinct disease phenotypes. Our results show that immunoprofiling is helpful for the evaluation of clinical prognosis in patients with CAC as well as sporadic CRC.

## Methods

### Study population

We enrolled patients with CAC and sporadic CRC who underwent surgery between September 1998 and December 2013 at Asan Medical Center, a tertiary university hospital in Seoul, South Korea. CD and UC were diagnosed by using conventional clinical, radiologic, endoscopic, and histopathologic criteria. Patients in the CAC group were diagnosed with colorectal adenocarcinoma in the chronic inflammatory lesions of underlying IBD during follow-up periods. CRC and UC were simultaneously diagnosed in two patients, in whom the CRC was found within the UC-involved area. Except for those two patients, the median duration from IBD diagnosis to CRC diagnosis in the CAC group was 14 years (range, 1–25 years). We included CAC patients from our previous study, which evaluated the incidence and risk factors of CRC in Korean IBD patients^20^. We selected sporadic CRC patients who were matched at 1:2 ratio according to age, sex, and disease location with CAC patients. Sporadic CRC group included patients who had undergone radical surgeries after being diagnosed with colorectal adenocarcinoma. We excluded patients with hereditary CRC such as familial adenomatous polyposis or hereditary nonpolyposis CRC, family history of CRC, or history of other malignant tumors. Clinical information of IBD patients was retrieved from the IBD registry of Asan Medical Center, which has held prospectively maintained computerized records of medical information including patient demographic and clinical information since 1997. Any changes in patients’ information such as progression of disease activity, development of complication, or start of new medication were updated at each clinic visit or at least yearly. The clinical and pathological information on sporadic CRC patients was also obtained from the medical records. Recurrence during follow-up periods was evaluated in patients with stages I-III at the diagnosis of CRC. The protocol of this study was approved by the Institutional Review Board of Asan Medical Center, Seoul, Korea (No. 2015-1172) and all methods were performed in accordance with relevant guidelines and regulations.

### Tissue preparation and multiplexed immunofluorescence

All tissues of CAC and sporadic CRC groups were acquired from surgical specimens, which were collected from patients on the basis of informed consent. As for five patients with CAC who had not completely undergone cancer surgeries, their samples were acquired when they underwent tumor excision or biopsy with palliative ileostomy. We reviewed the tissues of all patients with a pathologist (J.H.K.) and selected slides that appropriately showed center and peripheral characteristics of tumors.

Tissue samples were acquired as 4 µm-thick serial cuts from formalin-fixed paraffin-embedded (FFPE) blocks. After deparaffinization, the slides underwent five sequential rounds of multiplex immunohistochemistry as follows: blocking was carried out with antibody diluent (ARD1001EA, Perkin-Elmer, USA), followed by incubation with primary antibody, polymer HRP Ms + Rb secondary antibody (ARH1001EA, Perkin-Elmer) and Opal^TM^ tyramide signal amplification (TSA) plus agent. Specifically, antigen retrieval was carried out in 0.01 M citrate buffer (pH 6.0) using microwave treatment. Slides were cover-slipped using HIGHDEF^®^ IHC fluoromount (ADI-950-260-0025, Enzo, USA).

The primary antibodies and corresponding TSA used for each protein were as follows: anti-Foxp3 (236/E7, ab20034, Abcam, USA) and Opal 690 for Foxp3, anti-PD-L1 (E1L3N, #13684, Cell signaling, USA) and Opal 650 for PD-L1, anti-CD8 (4b11, NB100-65729, Novusbio, USA) and Opal 620 for CD8, anti-CD3 (2GV6, 790–4341, Ventana, USA, dilution) and Opal 540 for CD3, and anti-CK (AE1/AE3, M3515, Dako, USA) and Opal 520 for CK. Nuclei were visualized with DAPI. The methods have been introduced in further detail in a previous study^14^.

### Multispectral imaging and quantitative data analysis

Stained slides were imaged at 10X and 20X through the Vectra 3.0 Automated Quantitative Pathology Imaging System (Perkin-Elmer). The slides scanned at 10X (both multiplexed IHC and hematoxylin and eosin) were reviewed again by a pathologist (J.H.K.) to select sites that appropriately showed the invasive front, which were then scanned at 20X.

**Figure 5:**
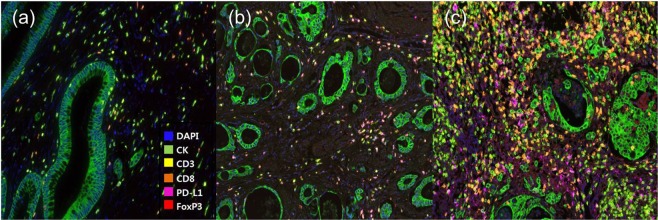
Representative images of multispectral immunohistochemical staining. (20X) (**a**) CAC in a patient with Crohn’s disease. (**b**) CAC in a patient with ulcerative colitis. (**c**) Sporadic CRC.

The magnified scanned image files were analyzed using Inform™ 2.2 image analysis software (Perkin-Elmer). The intensity of each fluorescent target was extracted from the multispectral data using linear unmixing. The spectral library was used as the reference for target quantitation. Figure 5 shows the representative images of multispectral immunohistochemical staining and Supplementary Fig. 1 shows the process of the multiplexed immunofluorescence imaging system. Each cell was identified by detecting nuclear spectra elements (DAPI). Each tissue was segmented into either tumors or stroma regions according to positive or negative staining of CK. Then, the cell segmentation of each cell was classified into nucleus, cytoplasm, and membrane. After accurate cell segmentation, we carried out cell phenotyping, in which each cell was chosen as a phenotype for the immune markers. The phenotyping feature was trained to choose cells with high confidence (≥60%). Besides phenotyping, we analyzed the expression of immune cells by the double positivity scoring method. This method categorized the cells into positive and negative intensity according to the threshold score evaluated with normalized count value for each antibody, provided by the Inform^®^ software. For the double positivity scoring analysis, we counted the number of cells simultaneously expressed two immune markers by exporting each marker’s score using the Spotfire^™^ program (Perkin-Elmer). Expression intensity was compared and then judged based on the cut-off value. The numbers per mm^2^ of CD3, CD8, Foxp3, PD-L1, and cytokeratin positive cells were counted in each slide.

### Comparison of conventional and multiplexed IHC staining

We selected 12 patients from each group to compare immune markers between conventional and multiplexed IHC. Multiplexed IHC staining was performed using Leica BondRX Automated Stainer (Leica Biosystems, Newcastle, UK) on a single sectioned slide, and conventional IHC staining was performed in five serial slides for five immune markers. The same antibody conditions were used in both multiplex and conventional IHC staining. Each cell was then scored by a range of standard pathology scoring systems (percentage positivity, H-score, 0/1+/2+/3+) with the H-scoring program embedded in the Inform^®^ software. The percentage of cells at each staining intensity level was calculated, and an H-score was assigned using the following formula:

$$1\times ( \% \,{\rm{cells}}\,1\,+\,)+2\times ( \% \,{\rm{cells}}\,2\,+\,)+3\times ( \% \,{\rm{cells}}\,3\,+\,).$$

### Statistical analysis

Baseline characteristics and clinical outcome parameters were compared between CAC and sporadic CRC. Comparison of immune cells between both groups was also carried out. All *p* values < 0.05 were considered to be statistically significant. Continuous variables were compared by Student’s *t*-test or Mann-Whitney U test, and categorical variables were compared with chi-square test or Fisher’s exact test. We evaluated the relationship between mortality and densities of CD3^+^, CD8^+^, Foxp3^+^, and PD-L1^+^ cells by receiver operating characteristic (ROC) analysis. The cut-off value of each immune cell was calculated by Youdan index J or closest top left criterion as appropriate. The immune markers of CAC and sporadic CRC had separate cut-off values because the distribution patterns of immune markers in the two groups were different. Overall survival (OS), defined as the time from the date of CRC diagnosis to the date of death or the last follow-up, was calculated using the Kaplan-Meier method. All deaths in this study were CRC-related. SPSS software (version 22.0; SPSS, Chicago, Illinois, USA) and R software (version 3.4.1, package ‘pROC’; R Foundation for Statistical Computing, Vienna, Austria) were used for statistical analyses.

## Supplementary information

Supplementary Table 1 and Figure 1
