# Supplementary material for: Immunoprofiling of Colitis-associated and Sporadic Colorectal Cancer and its Clinical Significance
Source: Sci Rep. 2019 May 2;9:6833. doi: 10.1038/s41598-019-42986-1 (PMC6497720; doi:10.1038/s41598-019-42986-1)

**Immunoprofiling of Colitis-associated and Sporadic Colorectal Cancer and**

**its Clinical Significance**

Jae Seung Soh, Su in Jo, Hyejin Lee, Eun-ju Do, Sung Wook Hwang, Sang Hyoung Park, Byong Duk Ye, Jeong-Sik Byeon, Suk-Kyun Yang, Ji Hun Kim, Dong-Hoon Yang, Sang-Yeob Kim, Seung-Jae Myung

**Supplementary Table 1. Comparison of immune cell densities between colitis-associated cancer patients and stage-matched sporadic colorectal cancer patients in stages I – III.**

|  | CAC (n = 16) | Sporadic CRC (n = 16) | *p*-value |
| --- | --- | --- | --- |
| CD3, mm^2^, mean (SD) | 421 (160) | 685 (438) | **0.032** |
| CD8, mm^2^, mean (SD) | 371 (146) | 613 (280) | **0.007** |
| Foxp3, mm^2^, mean (SD) | 121 (90) | 196 (92) | **0.007** |
| PD-L1, mm^2^, mean (SD) | 203 (220) | 998 (520) | **<0.001** |

**Supplementary Figure 1.** Results of multiplexed immunofluorescence imaging system. Multiple immuno-histochemical staining carried out on a slide of sporadic colorectal cancer tissue with different pseudocolors for each immune cell type (CK-green, CD3-yellow, CD8-orange, PD-L1-magenta, and Foxp3-red). After identifying with composite images to stain immune cells adequately, tissue segmentation, cell segmentation, and phenotyping were carried out for quantitative analysis.


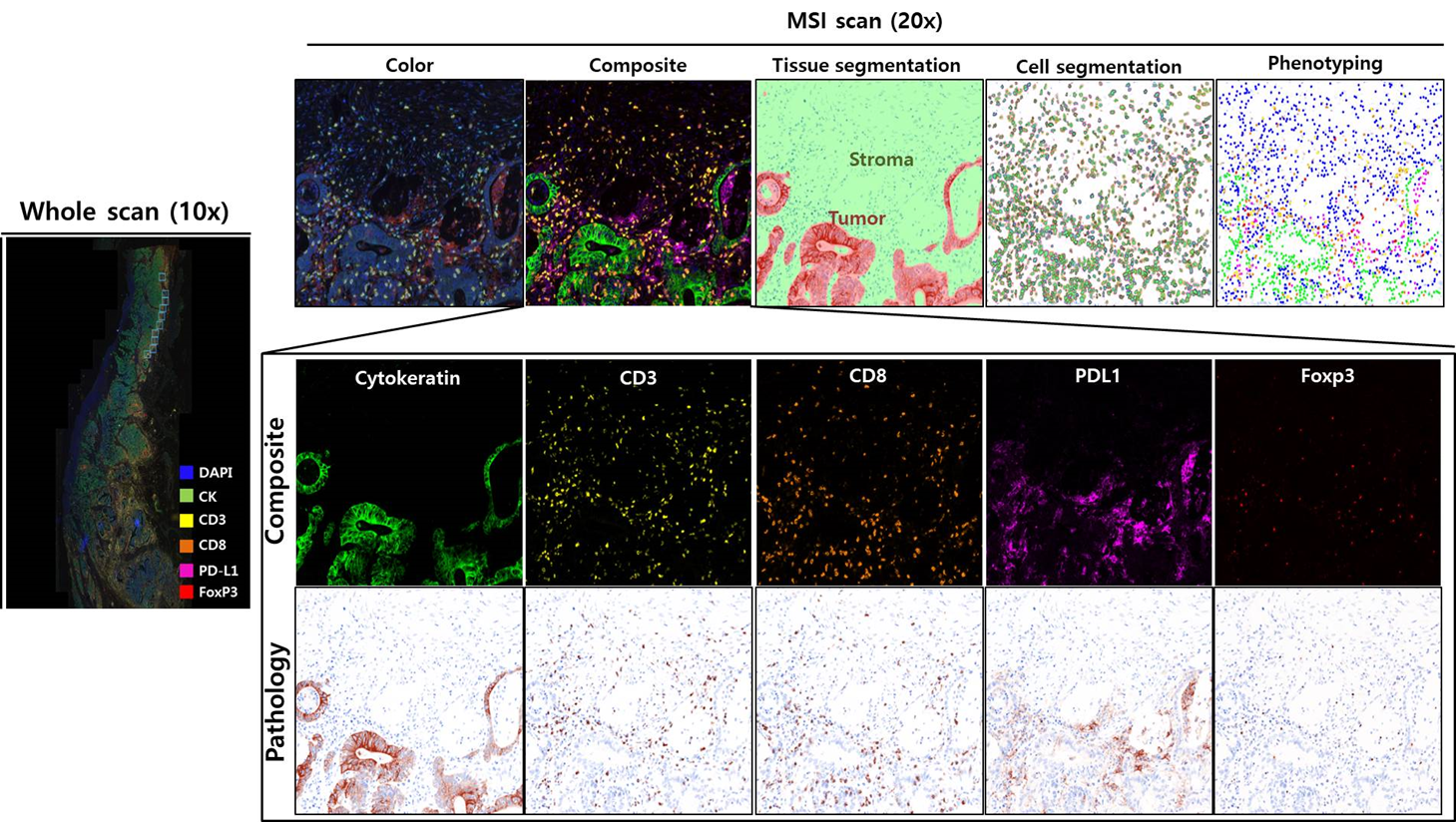

Supplement: Supplementary file 1 — Supplementary Table 1 and Figure 1 [file 41598_2019_42986_MOESM1_ESM.doc]
